# Hypothyroidism Among College Students and Its Association With Academic Performance: A Cross-Sectional Study

**DOI:** 10.7759/cureus.42588

**Published:** 2023-07-28

**Authors:** Rehab A Mohammed, Omar A Baqais, Samaher G Basalib, Abdulaziz Z Owaidah, Abdulrahman T Mirza, Intessar Sultan

**Affiliations:** 1 Department of Internal Medicine, Ibn Sina National College for Medical Studies, Jeddah, SAU; 2 Department of Internal Medicine, Faculty of Medicine for Girls, Al-Azhar University, Cairo, EGY; 3 Department of Medicine, Ibn Sina National College for Medical Studies, Jeddah, SAU

**Keywords:** and learning difficulties, gpa, grade point average, academic performance, hypothyroidism, thyroid

## Abstract

Background: Little is known about the impact of hypothyroidism and its contribution to learning difficulties and academic performance in college-age students.

Objective: The objective is toassess the frequency of hypothyroidism and its associations with academic performance in college-age students.

Methods: A cross-sectional study included 395 students studying across various Jeddah universities and selected by non-probability convenient sampling. Students self-answered the survey using Google Forms. The survey included demographic data, thyroid data, academic achievement as measured by overall grade point average (GPA), and student persistence as measured by academic failure and delay.

Results: Three hundred ninety-five students were included, their median age was 23 years (17-33), 96 were males (24.3%) and 299 were females (75.7%). Their median GPA was 4 (2.76-4). Thirty-two students (8.1%) had been treated for hypothyroidism and eight students (25.8%) were non-compliant with therapy. Odds of hypothyroidism increased among females (OR 3.33, 95% CI: 0.99-10.78, p=0.048), older age (OR 2.60, 95% CI: 1.33-5.77, p=0.009), those with a positive family history of thyroid illnesses (OR 5.49, 95% CI: 2.30-13.07, p<0.001), and those with academic failure (OR 3.31, 95% CI: 1.43-7.30, p=0.003) and academic delay (OR 2.83, 95% CI: 1.14-7.05, p=0.020). There was no significant association between hypothyroidism and GPA (OR 2.42, 95% CI: 0.83-7.77, P=0.092).

Conclusion: Hypothyroidism was prevalent among college students (8%), especially among older females. Hypothyroidism was associated with difficult student persistence, but this association did not reflect on their overall academic achievement. Incompliance with thyroid replacement therapy may be a common issue among hypothyroid patients. Further studies should focus on specific tests of the cognitive function of different learning domains and the role of treatment.

## Introduction

Poor health has a significant impact on student's overall performance and increases the likelihood of academic failure, grade retention, and dropout [1]. Many studies showed that grade point average (GPA) is associated with numerous physiological and psychological disorders [2,3]. However, few studies have been conducted among college-age students with hypothyroidism, a group that may be at elevated risk for academic difficulties.

Overt hypothyroidism can lead to a significant decline in mood and cognitive function including general intelligence, attention, memory, perceptual and psychomotor function [4]. Neuroimaging studies provided objective evidence of altered brain structure and function in hypothyroid patients with a reduction of hippocampal volume and cerebral blood flow [5]. In addition to cognitive impairment, fluctuations in the level of thyroid hormone may be manifested by somatic symptoms such as lethargy, fatigue, and sleep disorders that can adversely affect students’ interest in their studies [6].

Despite hypothyroidism being one of the most common endocrine disorders, there is a paucity of data regarding its prevalence in college-age students worldwide including Saudi Arabia. Moreover, it has not been studied if the extent of cognitive impairment in hypothyroidism is marked enough to affect the academic performance of college-age students. Therefore, this study was carried out to determine the frequency of hypothyroidism in college students and to determine the correlation between hypothyroidism and academic performance including academic achievement and student persistence.

## Materials and methods

Participants

A cross-sectional study was constructed to include college students studying at various Jeddah universities and colleges in different fields and specialties who agreed to participate in this study. Exclusion criteria were students with chronic illnesses such as diabetes, renal, or hepatic diseases and students under centrally acting drugs that can affect cognition.

Students were included in the study using a nonprobability convenient sampling. A minimum required sample size of 384 was estimated using Epi-Info version 7 based on a 5% margin of error, a design effect of 1, a cluster of 1, and an expected frequency of 50%. A data collection Google form was created in both Arabic and English languages and distributed via mobile applications. The questionnaire inquired about facts about their socio-demographics (age, gender), and current medical conditions.

The second part of the questionnaire included their academic data and academic performance. Their performance was assessed using their academic achievement operationalized as GPAs and their student persistence. Student persistence is considered one of the most important metrics in higher education. Student persistence measures the student’s activity and engagement. In the questionnaire, students were asked if they had experienced any of the following: failure (repeated courses, repeated years) or delay (postponing courses, changing courses or college, leaving college before completing their degrees, or transferring from one college to another).

All students were asked about their personal and family history of thyroid disease in the last part of the questionnaire. Students were classified into those with and without a history of hypothyroidism. For those with hypothyroidism, details of their hormonal replacement therapy (HRT) were included in the questionnaire including the dose and compliance with treatment.

Ethical consideration

Ethics and confidentiality of the data were assured. All participants gave informed written informed consent to participate in the study. The study was approved by Ibn Sina National College Institutional Research Review Board (IRB 001MP10102022).

Statistical analysis

Statistical analysis was conducted using SPSS statistics v. 22.0 software (IBM Corp., Armonk, NY), and a New Microsoft Excel sheet was used to construct the figure. The Shapiro-Wilk test was used to test the normality of the study samples and the median and IQR were chosen to describe variables with abnormal distribution. Students were categorized according to their average total GPAs. The GPA between 3.0 and 5.0 is deemed to be average to high and below 3.00 is low. Simple frequency tables were used for the descriptive analysis of categorical variables. Chi-squared test was used for comparison with an estimation of the odd ratio, when possible, with a 95% confidence interval (95% CI). The level of significance for the study was set at <0.05.

## Results

**Figure 1:**
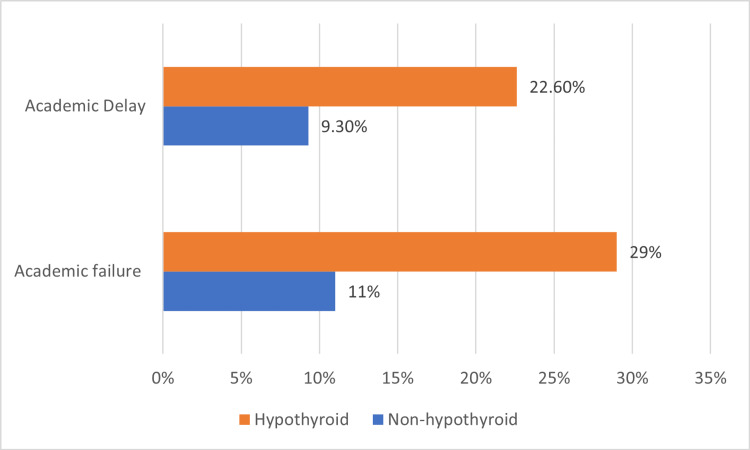
Comparison between students with and without hypothyroidism regarding their academic persistence

The study included 395 students with a mean age of 23 (IQR 3) years. Female students accounted for 75.7% of them and 269 (68.1%) were from the health field studies. The students' median overall GPA was (3.73 ranging from 2.76 to 4. Low GPA was found in 100 students (25.3%) (Table 1). For student persistence, academic failure was reported in 49 (12.4%), while 41 (10.4%) had an academic delay (Table 1, Figure 1).

**Table 1:** Characteristics of the participating students GPA: Grade point average

	N= 395 N		%
Gender			
Female	299		75.7
Male	96		24.3
Age: median (IQR): years	23 (3)		
Age groups			
<=23	262		66.3
>23	133		33.7
Nationality			
Non-Saudis	84		21.3
Saudis	311		78.7
Specialty			
Health field	269		68.1
Other fields	126		31.9
GPA: median (IQR)	3.76 (1)		
GPA categories			
Low	100	25.3	
High	295	74.7	
Failure (Repeated courses, repeated years)			
No	346	87.6	
Yes	49	12.4	
Delay (postponing/ changing, transfer)			
No	354	89.6	
Yes	41	10.4	

History of treated hypothyroidism was identified in 31 (8%); however, eight of them were noncompliant (25.8%) to treatment. Positive family history of thyroid disorders was found in 164 (41.5%) of all participants and in 24 (77.4%) of those with hypothyroidism. Three students had hypothyroidism post-thyroidectomy (9.7%) and four post-radioiodine therapy (12.9%). The median duration of illness was two years (IQR 4) with a median dose of 50 microgram daily (IQR 69) (Table 2).

**Table 2:** Characteristics of hypothyroidism among participating students HRT: Hormonal replacement therapy

	N=393	%
Hypothyroidism	32	8.1
Family history of thyroid disease	24	77.4
Known causes of hypothyroidism		
Post surgery	3	9.1
Post-radioiodine	4	12.1
Compliance to HRT		
Complaint	24	74.2
Non-compliant	8	25.8
Dose of HRT: median (IQR): microgram/day	50 (69)	
Duration of disease: median (IQR): years	2 (4)	

Hypothyroid patients had significantly increased odds of being females (OR 3.33, 95% CI: 0.99-10.78, p=0.048), older age (OR 2.60, 95% CI: 1.33-5.77, p=0.009), had positive family history of thyroid illnesses (OR 5.49, 95% CI: 2.30-13.07, p<0.001). Hypothyroid patients also had significantly increased odds of having academic difficulties including failure (OR 3.31, 95% CI: 1.43-7.30, p=0.003) and delay (OR 2.83, 95% CI: 1.14-7.05, p=0.020). There was no significant association between hypothyroidism and GPA (OR 2.42, 95% CI: 0.83-7.77, P=0.092) (Table 3).

**Table 3:** Comparison between students with and without hypothyroidism

		Hypothyroid				P	OR	95%CI		
		No N=364		Yes N=31						
		N	%	N	%			Lower		Upper
Age	<=23	248	68.1	14	45.2	0.009	2.60	1.33	5.77	
	>23	116	31.9	17	54.8					
Gender	Female	271	74.5	28	90.3	0.048	3.33	0.99	10.78	
	Male	93	25.5	3	9.7					
Nationality	Non-Saudi	76	20.9	8	25.8	0.520	1.32	0.57	3.06	
	Saudi	288	79.1	23	74.2					
Family history of thyroid illnesses	No	224	61.5	7	22.6	<0.001	5.49	2.30	13.07	
	Yes	140	38.5	24	77.4					
Specialty	Non-health field	117	32.1	9	29	0.721	1.16	0.446	2.50	
	Health-field	247	67.9	22	71					
GPA	Low	96	26.4	4	12.9	0.092	2.42	0.83	7.77	
	High	268	73.6	27	87.1					
Academic Delay	No	324	89	19	71	0.003	3.31	1.43	7.30	
	Yes	40	11	9	29					
Academic Failure	No	330	90.7	24	77.4	0.020	2.83	1.14	7.05	
	Yes	34	9.3	7	22.6					

## Discussion

In this study, 8% of college students in Jeddah, SA, had hypothyroidism under treatment with a quarter of them incompliant with treatment. They were mostly females. The hypothyroid condition was associated with lower student persistence including higher academic failure and delay. There was no significant association between the disease and academic achievement.

The population-wide prevalence of hypothyroidism in SA is not well studied especially among different age categories. One study reported undiagnosed hypothyroidism in 5.5% of participants [7]. The prevalence of subclinical hypothyroidism reached 10.3% in one primary care center study in Riyadh [8]. However, higher prevalence rates were reported in other studies in the kingdom exclusively in women. From Arar, a prevalence rate of 25.5% was reported in those aged 21-60 years old [9]. The highest prevalence (35%) was reported in those aged above 50 years [10] reflecting age as a contributing factor in elderly patients. Hypothyroidism reported in a study from the Hail region was extremely high as 56% was conducted at an endocrine clinic [11]. While a study on thyroid diseases in the Arab world reported a prevalence of 47.34% in SA [12].

Primary hypothyroidism could occur at any age, but it peaks in the fifth decade [13]. The prevalence of hypothyroidism in this study is much lower than other reported frequencies in SA probably because of the selected young age group of our college students and the inclusion of hypothyroid patients under treatment. Our result is in line with a study conducted among 100 medical students in India who reported that 8% of their students had subclinical hypothyroidism [13].

Hypothyroidism is a common chronic illness, especially among women that is amenable to therapy. Untreated hypothyroidism can contribute to cognitive impairment [14]. Ettleson et al. [15] developed a survey to determine cognitive and behavioral symptoms associated with hypothyroidism and they found that more than 95% of the participants had fatigue, forgetfulness, feeling sleepy, and difficulty focusing, all these combined symptoms have negative impacts on the life and decrease the neurocognitive reserves that may affect the learning ability. Hrytsiuk et al. [16] assessed the mean intelligence quotient (IQ) of 30 students who are treated for hypothyroidism and they found that their IQ scores were within the normal range. However, 77% showed at least one sign of impaired brain function and 26% of them had learning disorders. This is contrary to Najmi et al. [17] who found that mean IQ scores in transient congenital hypothyroidism and permanent congenital hypothyroidism was lower than the control group.

In this study, although the hypothyroid condition is associated with lower student persistence, the differences in the student's GPA did not reach statistical significance. This discrepancy may be explained that overall GPA is not a consistently accurate measurement of learning [18]. It could be explained also by being euthyroid by HRT as 75% of students were compliant with treatment. These results highlight the importance of screening college students for hypothyroidism for better academic performance. However, all currently available population screening recommendations involve adults far above college age; from 35 years [19], elderly [20], or those with known risk factors [21].

Limitations

This study has several limitations. First, the cross-national prevalence estimate was based on a non-randomized convenience sampling of college students that limited the generalization of the results. Second, the number of hypothyroid students was small which limited the statistical analysis of the possible association between duration, dose, and compliance with academic performance. Third, the study included self-reported academic data which might be subjected to denial or overestimation. Although it would have been desirable to include a more objective assessment, this might be difficult to gain approval from different college administrations.

## Conclusions

We conclude that despite the limitations of the cross-sectional study of the selected age group, hypothyroidism is prevalent in 8% of college students. The majority of our patients were predominantly females aged above 23 years. These two observations should be further validated by population-based studies for the true frequency nationally. Incompliance with thyroid replacement therapy may be a common issue among hypothyroid college students. Hypothyroidism is associated with lower academic performance including failure and delay. These findings may support screening for thyroid dysfunction in the context of learning difficulties. Further studies using specific tests to evaluate the cognitive functions of different domains and the role of HRT are needed.
